# HOW DOES THE INTERSECTION OF RACE AND GENDER AFFECT HOSPICE CARE AT THE END OF LIFE?

**DOI:** 10.1093/geroni/igac059.3082

**Published:** 2022-12-20

**Authors:** Zainab Suntai

**Affiliations:** Baylor University, Waco, Texas, United States

## Abstract

Research in the palliative and end-of-life-care field has shown that hospice care in the final months of life can improve outcomes such as pain, emotional well-being, and physical comfort. Yet, researchers often find significant disparities in the ability to access hospice care, with Black individuals being less likely to have hospice care at the end of life. The theory of intersectionality suggests that the combination of multiple vulnerable identities may add to the number of hardships and stressors that an individual experiences across the lifespan. To test the theory, this study aimed to access whether gender moderated the relationship between race and the receipt of hospice care at the end of life. Data were derived from Round 3 to Round 10 of the National Health and Aging Trends Study, and two multivariate regression models were used to assess the relationship between the race/gender interaction and hospice care at the end of life (Model 1: main effects, Model 2: interaction term). Results showed that the effect of race on hospice care was indeed dependent on gender, with Black women being the least likely to have hospice care at the end of life. This points to the combined disadvantages resulting from membership in two vulnerable groups (i.e., being a woman and being Black), including fewer hospice facilities in Black-populated areas, race and gender discrimination in hospice referrals, and other factors that combine to reduce hospice access for Black women. Implications for research, practice, and policy are provided.

